# Factors affecting retention of allied health professionals working with people with disability in rural New South Wales, Australia: discrete choice experiment questionnaire development

**DOI:** 10.1186/s12960-015-0013-7

**Published:** 2015-04-21

**Authors:** Gisselle Gallego, Angela Dew, Kim Bulkeley, Craig Veitch, Michelle Lincoln, Anita Bundy, Jennie Brentnall

**Affiliations:** Centre for Health Research, School of Medicine, University of Western Sydney, Building 3, Campbelltown Campus, Locked Bag 1797, Penrith, New South Wales 2751 Australia; Faculty of Health Sciences, University of Sydney, Cumberland Campus, East St, PO Box 175, Lidcombe, New South Wales 1825 Australia; Faculty of Arts and Social Sciences, University of New South Wales, Sydney, New South Wales 2052 Australia

**Keywords:** Discrete choice experiment, Allied health professionals, Preferences, Retention, Rural, Disability

## Abstract

**Objective:**

This paper describes the development of a discrete choice experiment (DCE) questionnaire to identify the factors (attributes) that allied health professionals (AHPs) working with people with disability identify as important to encouraging them to remain practising in rural areas.

**Methods:**

Focus groups and semi-structured interviews were conducted with 97 purposively selected service providers working with people with disability in rural New South Wales, Australia. Focus groups and interviews were digitally recorded, transcribed, and analysed using a modified grounded theory approach involving thematic analysis and constant comparison.

**Results:**

Six attributes that may influence AHPs working with people with disability in rural areas to continue to do so were inductively identified: travel arrangements, work flexibility, professional support, professional development, remuneration, and autonomy of practice. The qualitative research information was combined with a policy review to define these retention factors and ensure that they are amenable to policy changes.

**Conclusion:**

The use of various qualitative research methods allowed the development of a policy-relevant DCE questionnaire that was grounded in the experience of the target population (AHPs).

## Introduction

Shortages of allied health professionals (AHPs) in rural and remote areas are widespread in Australia and are more significant in areas of special need, such as mental health, aged care, and disability services [1-3]. Compared to their medical counterparts, larger numbers of rural AHPs are leaving the workforce by moving to other jobs, reducing their participation by going part-time or casual, or retiring [4]. In Australia, an important transition is about to occur in the systems that provide people with disability access to therapy nationally with the introduction of the National Disability Insurance Scheme (NDIS). Labelled as the biggest social reform since the introduction of Medicare (universal health care), the NDIS will “*support choice for people with disability*, *their families and carers*, *and put people in control of the care and support they receive*, *based on need*” [5]. However, as the NDIS is rolled out, people with disability living in rural areas are vulnerable to a loss of access to allied health services due to shortages of AHPs and increasing demand for their services. AHPs, including speech pathologists, occupational therapists, physiotherapists, and psychologists are key service providers to people with disability. These AHPs optimize functioning and independence for individuals and ensure they can participate as fully as possible in their communities [6]. Even though the workforce has grown at a faster rate than the population, there is still a difference between the number of AHPs per 1 000 head of population in rural versus metropolitan areas [7]. However, the Australian Government, by allowing unlimited numbers of allied health university courses and students, as well as various workforce initiatives, has rapidly increased the size of the allied health workforce. For example, the physiotherapy workforce in remote areas in Australia grew by 23.8% between 2011 and 2012 [8,9]. The problem is now about availability of positions, distribution, and retention of the workforce in rural areas [7].

Research has also shown that a range of recruitment strategies (salary packages, moving expenses, choice in location) that are in place appeared to have worked in attracting AHPs. Nevertheless, there are difficulties keeping AHPs in those jobs (i.e. retention) [10].

Besides the introduction of the NDIS, demand for therapy services will increase significantly due to the ageing of Australia’s population, increased life expectancy, demography, and population growth [11-13]. The supply of AHPs in rural and remote areas with the experience and necessary skills may not meet the increased demand. It is important to understand the factors that may encourage AHPs to remain working with people with disability in rural and remote areas.

In recent times, discrete choice experiments (DCEs) have been used to study the preferences of health workers [14,15] and to provide insight into potential policy responses to the problem of rural retention [16,17]. DCEs have also been used to explore how health workers would respond to incentives associated with working in a rural setting [15,17,18]. DCE methodology is a process that determines the comparative or relative value that people place on a set of factors (attributes). The method’s particular strength is that it determines what people are prepared to forego in order to maintain or obtain something else [19].

In a DCE design to explore job choices, for example, a hypothetical job is described by a number of attributes (e.g. work location, pay levels, access to professional development), each of which is described at varying levels. Several possible jobs—combinations of different attributes at different levels—are presented to each respondent and they then choose which of the jobs they prefer. Each such set of possible jobs is termed a choice set, and the DCE consists of obtaining the respondents’ preferences for each of the choice sets. This allows modelling of respondents’ preferences, including how they trade-off different job characteristics. It can also explore how these trade-offs change over time in response to their actual experiences. Information on the relative importance of the selected attributes is useful for policy makers. DCEs can provide an indication of the level of uptake that could be expected given a particular policy criterion (i.e. different types or levels of payment to influence retention) and the impact of workforce socio-demographic characteristics [20]. For example, Mangham and Hanson [18] explored the employment preferences of public sector nurses in Malawi and the trade-offs between non-monetary benefits (e.g. provision of government housing) and working conditions (e.g. place of work: rural versus urban).

DCEs offer an opportunity to study the determinants of AHPs’ job preferences, serving as a valuable tool to inform decision-makers on how to design effective strategies to retain them [17]. To date, no study has explored the preferences of AHPs or health professionals working with people with disability. While some of the issues faced by medical practitioners and nurses could be similar to those faced by AHPs, the nature of the allied health professions and their job characteristics need further exploration. The characteristics and motivations of AHPs working with people who have lifelong conditions are very likely to be different from those health professionals who more often work intermittently with patients who are free of long-term conditions. Furthermore, evaluating policy initiatives for retention in rural and remote areas is hampered by the dearth of generalizable, empirical data on AHPs’ behaviours, the determinants of their choices, and the implications of these dynamics in terms of policy.

This paper describes the development of a DCE questionnaire to identify the factors (attributes) that AHPs working with people with disability identify as important to encourage them to remain working and practising in rural areas. This formed the basis of a DCE embedded in a survey to understand the relative importance that AHPs working in disability services in a region in western New South Wales (NSW), Australia, place on different work characteristics, and the trade-offs they are willing to make between components, in order to ascertain their preferences.

## Methods

### Setting

With a population of 7.29 million, New South Wales (NSW) is the most populated Australian state [21]. The study took place in western NSW, which accounts for 72% of the land area and 8.2% of the state’s population [22]. The population in this region is scattered among large regional towns with populations of 20–40 000 mid-sized regional towns with 4–19 000 smaller towns of 1–3 000 people and isolated rural communities of less than 1 000 people. Some people live on remote farms hundreds of kilometres from towns [22].

### Sampling and recruitment

To identify a sample that would serve the purpose of the study and maximize the insights gained from qualitative analysis, purposive [23] and snowball [24] sampling techniques were used to identify the study participants. Inclusion criteria were as follows: i) allied health professionals: speech pathologists, occupational therapists, physiotherapists and psychologists, and other professionals working alongside AHPs: early childhood workers and behaviour support workers; ii) role: direct services provider, case managers, and line managers (with/without) a formal AHP training in the above mentioned fields; iii) being employed in a management or direct service role in a specialist government or a non-government agency (NGO) or privately employed; and iv) providing services to people with a disability who lived in western NSW. Further, theoretical sampling [23] strategies were used to ensure participants represented a diverse range of experiences based on differences in gender, age, geographic location, type of organization, and employment role. To recruit these participants, the investigators approached specialist government and NGOs providing services to people with a disability and their careers in western NSW. The nature of the study was explained to representatives of these organizations, and they were asked to pass on an invitation letter and information about the study to appropriate members of their organizations. The specialist government organization disseminated information sheets and consent forms to employees working in community access teams. Information about the study was also disseminated to NGO employees known to deliver services to people with a disability in western NSW. The majority of these organizations received funding from the specialist government organization. Staff who were interested and willing to participate then made contact with the research team.

### Data collection

Data were collected through focus groups and, for those unable to attend focus groups, individual interviews. A semi-structured focus group/interview guide was developed based on policy documents, literature review, and consultations with policy makers and senior staff members undertaken in an earlier stage of the study [25,26]. To encourage participants to share their experiences, participants were asked open-ended questions about a range of topics related to therapy service provision including the workforce issues impacting on allied health employment and service delivery to people with disability in a rural area (e.g. what variables impact on decisions to live and work in the area?). The guides facilitated group and individual reflection and discussion about a variety of workforce practices and challenges. Using the principle of theoretical sampling, emerging issues from one focus group or interview were further explored in subsequent groups and interviews until theoretical saturation was reached and no new issues emerged [24].

All data were collected by authors AD and KB, between March and July 2011. The focus groups ran on average 2 h and the individual interviews 1 h. A running summary of the overall impressions and record of the emerging themes during the focus groups was displayed during each focus group. This allowed participants to follow the flow of discussion and provided a prompt for further discussion. Baseline characteristics were collected using a brief demographic questionnaire on gender, age range, marital status, children, time living in the area and working in the current position, employer, previous experience in the disability field, time in the field, and qualification. All focus groups and interviews were digitally recorded with the consent of participants and then transcribed.

### Data analysis

A modified grounded theory approach, using thematic analysis and constant comparison, was used to analyse transcript data [27]. After preliminary analysis was performed, segments (paragraphs or sentences) were coded and labelled. Coded segments were then compared for differences and similarities in events and ideas. This process was repeated until all comments were assigned to categories (constant comparison) [28]. AD conducted the initial analysis, and in order to verify the emerging themes, KB performed an analysis check on a randomly selected 10% of the transcripts. Emerging themes were then discussed with the other authors until a consensus on the themes was reached. A summary of the themes was sent to participants with an invitation to provide additional comments or suggest changes. No amendments were requested by participants.

### Ethics

This study was approved by the Human Research Ethics Committee of The University of Sydney (#10-2009/12194) and the University of Western Sydney (H9446). Written consent was obtained from all study participants. All transcripts were anonymized, and all data were kept confidential.

## Results

### Description of participants

Eleven focus groups involving 92 participants (the size of the groups ranged from 2 to 16 participants) and 5 additional individual interviews were held across 13 geographic locations in western NSW to ensure that all participants had access to a group or interview without unreasonable travel. Table 1 summarizes the socio-demographic information provided by the participants. While the participants were encouraged to complete the demographic questionnaire, 15 chose not to do so. The majority of respondents were female (88%), aged 31 to 40 years (37%), in a married or *de facto* relationship (73%), and more than half (60%) had children.

**Table 1:** **Socio-demographic information of participants**

**Characteristic (** ***n*** **= 82)** ^**a**^	***N*** **(%)**
*Sex*	
Female	72 (88)
*Age*	
20–30	22 (27)
31–40	30 (37)
41–50	20 (24)
51–60	8 (10)
61 and over	2 (2)
*Family*	
With partner	60 (73)
Children	49 (60)
Mean number of children	1.5
*Born in a regional/rural remote area*	
Yes	59 (72)
*Employment status*	
Full-time	53 (65)
Part-time	29 (35)
*Previous experience in the disability field*	
Yes	55 (67)
*Employed as*	
Allied health professional	33 (42)
Manager	17 (21)
Case manager	18 (23)
Other^b^	11 (14)
*Employer*	
Specialist government organization	28 (34)
Specialist NGO	39 (48)
NSW Health	11 (13)
Other^c^	4 (5)
*Why live and work in western NSW (N = 81)*	
Past connection to WR	37 (45)
Partner’s work	10 (12)
Tree-change^d^	7 (9)
Job opportunity	18 (22)
Family	9 (11)
Time in WR (mean years)	21
Length of time in current job (mean years)	5
Length of time working in disability (mean years)	10

WR, western region.

^a^Fifteen participants did not complete the background information sheet.

^b^Other included: therapy assistant, behaviour support worker, teacher.

^c^Other included: education, human services.

^d^Move from cities to rural communities.

The findings are presented, first, in terms of the important factors described by the participants. The second section of the results explains the iterative process and how the DCE attributes and levels within the attributes were developed. To indicate the range of geographic locations of respondents, the following identifiers are used: LRT (large regional town with population of 20–40 000) or MRT (mid-sized regional town with population of 4–19 000). The number that follows this indicates the different towns represented.

### First iteration: key emerging themes

Preliminary analysis of the data identified eight factors: travel burden, work flexibility, professional support, professional development, opportunities for teamwork, career progression, autonomy of practice, and remuneration and incentives.

#### Travel burden

As previously noted, the density of the population in the research area varies enormously, and geography has a significant impact on the nature of the region and work conditions. AHPs often have to travel long distances to see clients. While accepted as part of the job, travel was regarded by participants as a burden encompassing two dimensions: distance and time away from home.

So you’re looking at a day’s travel, a day’s actual on-the-ground work and then another day’s travel, so the time management side of that [MRT1].

Another participant spoke about how distance impacts on the supervision provided to new graduate AHPs:

I could be responsible for support and supervision at a distance for somebody who’s quite new and is just starting up…it’s so overwhelming…so isolated and [senior colleagues] are that far away and when they do need support, it’s over the phone and it’s not that face-to-face contact [LRT3].

Overnight stays were seen as particularly problematic for the predominantly female workforce, many of whom had other caring responsibilities, such as for young children. One participant summed this up:

And so it’s not sustainable too when you do have a predominately female workforce and therapists [AHPs] in this region are young and we’ve all recently been married, what happens to those clients out west when we have babies? It’s not a sustainable service delivery [LRT1].

#### Work flexibility

Work flexibility was highlighted by participants as important in keeping AHPs in their jobs. Work flexibility included variable work hours (i.e. the ability to change start and finish times) and family-friendly conditions including time-in-lieu arrangements and the ability to take time off to attend to caring responsibilities. Participants valued flexible work conditions that allowed them to accommodate their personal circumstances. Flexibility was identified as important for both recruitment and retention.

I was told yes, we’re family friendly, we’re flexible, you’ll get two flexi days a month. I did enquire and then when I got the job, I found out that no, it doesn’t exist [MRT1].

#### Professional support

Professional support from colleagues and peers was recognized by participants as a major contributor to retention. Professional support included the ability to “bounce ideas” around with a colleague, de-brief after a stressful client interaction, or simply engage in collegial discussions.

We had a travel ban here a while ago, where we weren’t allowed to travel for budgetary reasons. It was very isolating, because we use our meetings, our regional meetings, as professional development. It’s really isolating when you can’t go [MRT2].

Professional support was reported as particularly important for less experienced AHPs or those new to the rural environment. One participant said:

I guess that’s one of the harder things for a new graduate in rural areas is that you don’t have that support. It’s really hard to develop your skills and probably a little slower to develop them [LRT3].

Working as a sole practitioner without ready access to colleagues and peers resulted in professional isolation. This was identified by participants as a particular challenge for AHPs working in the disability sector in rural areas, as explained by this participant:

There were no other therapists [AHPs] on-site, like there’s a visiting speechie [speech pathologist] and they’ve occasionally employed a physio [physiotherapist] to do some hours, but there’s none of that coming back from a visit and going, “I need to talk about this, I don’t know what to do here” [MRT3].

#### Professional development

Participants spoke about professional development as access to opportunities to enhance their clinical and professional skills via formal training and on-the-job learning. Access to professional development was identified by participants as an important factor in satisfaction with their employment and hence them remaining in the job. This exchange among government employees in one focus group highlighted the value placed on access to continue professional development (CPD):

And also I think the level of PD is fantastic.

Oh, excellent, it’s really good.

As opposed to lots of other places.

Which has definitely improved with [government initiative].

The PD that we get here has been unbelievable [LRT2].

From a therapy perspective, some of the therapists [AHPs] here have said one of the great benefits of working for [government department], and not being in private practice, is the professional development opportunities they have and the peer support. So I think in terms of [government department]…you’re part of a collegiate workforce and you can support one another, because that’s obviously the issue with private practice, unless you can develop those networks, you really rely on yourself and the luxury of going and training, it’s an expense, it means you can’t work for that time and all of those things [MRT1].

#### Opportunities for teamwork

Adopting a hub-and-spoke model means that AHPs can be situated together in teams and provide outreach to outlying areas. AHPs employed by government and some of the larger non-government organizations work in multidisciplinary teams. Participants were aware that transdisciplinary work (where team members overlap and cross over traditional roles with boundaries between the roles of team members blurred) is supported by evidence. However, due to the shortage of AHPs in rural areas, they reported little opportunity to work in that way as described by this participant:

We’ve had all this influence of transdiscipline; you know the big community therapy conference two years’ ago was all about the importance of transdisciplinary work. We’ve got lots of frustration and I think morale’s dipping because we don’t have the staff across the region to do that [LRT3].

#### Career progression

Within the government sector in particular, AHPs gain experience through acting in higher positions as described by this participant:

Everyone sort of acts up and then so a grade one from [town] was acting up as a grade three for the region, so her position became available but because the original acting grade three [AHP] was acting in another position she was holding up recruitment, so there was sort of like a four-step process. And no wonder people act up, like, why wouldn’t you act up? [LRT1].

Opportunities for career progression often resulted in AHPs leaving their clinical roles for managerial positions as described by this participant:

So you can’t keep the experienced people in the therapy roles…because it’s more attractive to work elsewhere [MRT4].

#### Autonomy of practice

In the broad sense, autonomy refers to “*the freedom to make choices and to have self*-*regulation in the pursuit of self-selected goals*” [29]. While autonomy is a broad term, participants described “autonomy of practice” as being able to use “clinical judgement”, as noted below. It focused on AHPs’ ability to provide care for their clients according to their professional judgement. AHPs are university-trained professionals who in their interventions with clients and interactions with other professionals are expected to exercise professional and clinical judgement. However, in rural areas where demand for therapy services outstripped supply, participants described client allocation and prioritization systems that undermined their capacity for autonomy in clinical decision making. In these systems, decisions about when, for how long, and by whom a client would be seen were made by managers, with impacts described by these participants:

And it’s very frustrating and upsetting for therapists [AHPs], I think, who can see a family in need and be restricted by policy and just not to be able to provide that [MRT3].

So the actual worker has no autonomy over what they want to do with the client and if you went out and saw the client and went, “Oh, actually they need this”. “No, sorry, you have to do what’s in the referral” [LRT4].

I think they [specialist government agency] are very rigid. I think [the client allocation system] was breeding inflexibility and a slight preciousness. One day where the [AHP] came and couldn’t see any of the people who were allocated…it would have been good to pick up somebody else, [she] didn’t actually then think “well there’s 14 on the waiting list, I’ll just pick up the next one”. It was such a waste of a flight [MRT2].

#### Remuneration and incentives

The final theme that emerged from the first iteration was remuneration and incentives. Participants reported varied pay scales depending on the sector in which they were employed (i.e. health, disability, education). While there are set pay levels within different sectors employing AHPs, participants appreciated that incentives could offset some of the discrepancies in remuneration which occur for AHPs working in the government versus the NGO sectors.

So why don’t we have one industrial tool for allied health for all government agencies across the state? That’s the question, because it shouldn’t matter what agency really, we should use the same industrial instrument [LRT1].

### Second iteration: clarification and confirmation of attributes

In the second iteration, further analysis was undertaken on the frequency with which each factor was raised by participants, the perceived impact of each factor on retention, and how each factor could be described and presented in the format of a DCE. This was a two-step process: 1) confirming which themes would be most appropriate for inclusion as attributes in the DCE questionnaire and 2) refining the description of the attributes and attribute levels.

### Step 1—Identifying retention factors from themes to DCE attributes

The themes described above, the policy document review [25,30], and the feedback from the project management and study reference groups were considered in this step. The project management and study reference groups were set up at the beginning of the project to monitor the project progress and provide stakeholder input and advice. The project management group included senior government managers from the study site, while the study reference group included middle managers and AHPs from government and non-government agencies and carers of people with a disability. The input of these group members helped the authors to consider which job attributes were amenable to policy changes. Six factors were identified as potentially of greatest importance to the retention of AHPs working in the disability sector in rural areas. These six factors became the DCE attributes: 1) travel arrangements, 2) flexibility in work hours, 3) professional support, 4) access to professional development, 5) autonomy of practice, and 6) remuneration.

The steps in the development of themes into DCE attributes is described here, illustrated with examples from remuneration and travel arrangements. As previously noted, the variation in pay and conditions based on workforce sector was raised by many participants. Participants reported that AHPs employed by the government to work in specialist disability services were paid more than their peers employed in the specialist disability non-government sector, but less than their peers employed in the generic NSW Health sector. Pay (and conditions) was identified by participants as a factor that led AHPs to move from one sector to another and from rural to urban settings.

While “higher salaries” was seen as a significant factor influencing retention decisions among AHPs working with people with disability in rural areas, “financial incentives” was also highlighted as a factor that may help people stay in a rural job. The variation in wages according to the job characteristics, sector, and type of employer (health versus disability; NGO versus government) meant that options such as change in income per year (after tax), total monthly income, and increase in salary were not appropriate. These were not feasible options that could be implemented for all AHPs across the rural area or amenable to policy change. Consensus was reached that “rural salary loading above current salary” was a feasible option. This is an alternative that all respondents to the DCE could relate equally to. Furthermore, rural loading and rural isolation payments are being considered for medical practitioners and dentists who practice in rural areas of Australia to ensure competitive remuneration.

Even though some participants mentioned career progression, this was not mentioned as often as the other factors. Hence, it was not included as one of the attributes in the DCE questionnaire.

The next step in the process was to identify which particular aspect of the attribute should be included in the DCE. The most salient aspect needs to be identified before it can be operationalized (e.g. financial incentives were chosen above before determining how to word that and choosing the levels). Travel is an important factor in rural and remote Australia, for example, and participants raised a range of issues related to travel including administrative requirements, fatigue, occupational health and safety issues, and disruption to family and social life. By returning to the analysis of the focus group and interview data, authors identified that the single most important travel-related factor to interviewees and focus group participants was the requirement to undertake travel that involved staying away from home overnight. Therefore, the travel DCE attribute focused on travel arrangements related to nights away from home. A similar process was applied to each of the other five identified attributes to determine the most salient factor which had potential to be modified to impact on retention.

**Figure 1:**
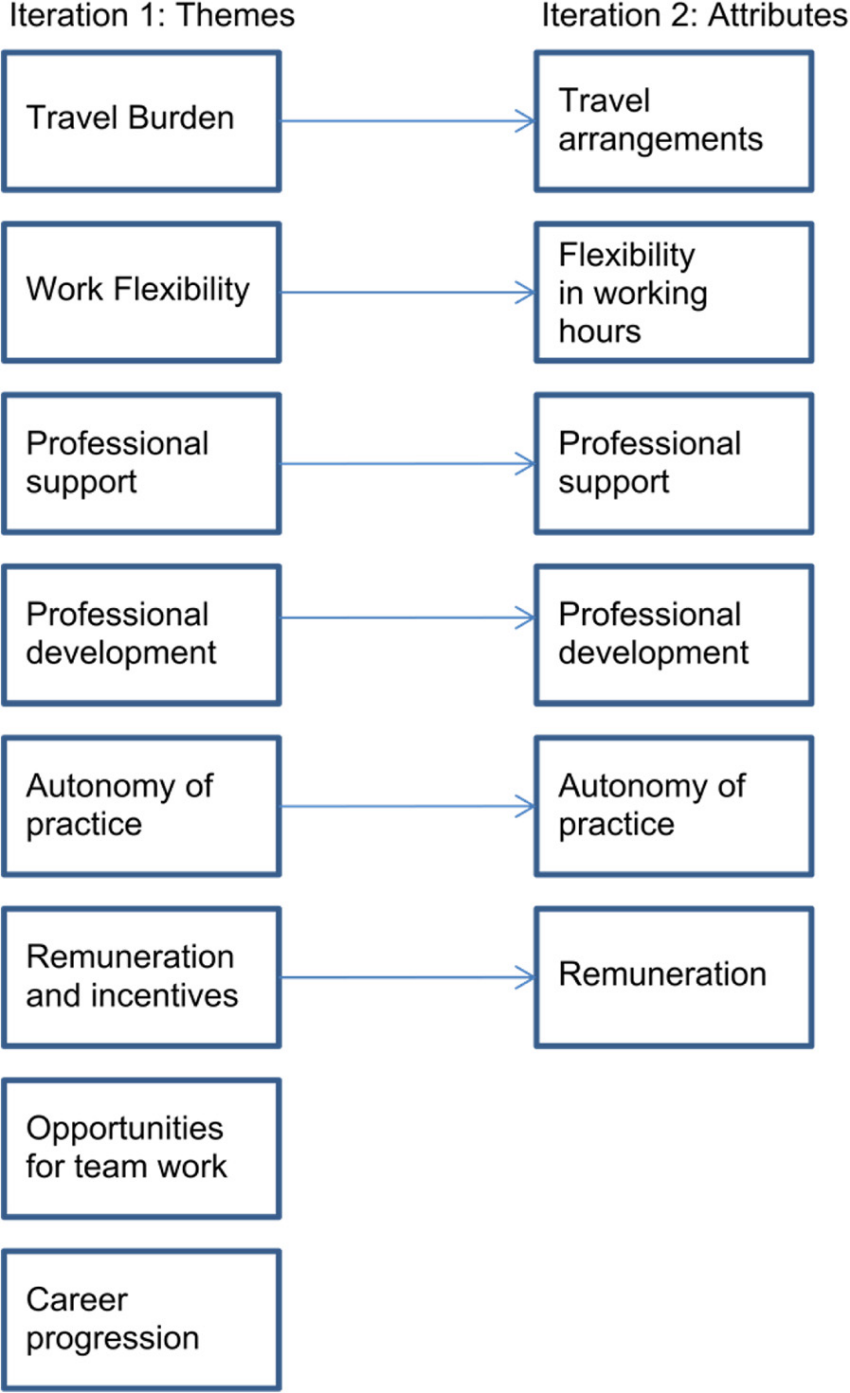
Attribute development process.

The process also allowed us to minimize inter-attribute correlations. For example, it was not appropriate to include both “travel distance” and “travel time” since they are closely related. Similarly, professional support and opportunities for teamwork were closely related, and the latter was chosen to drop from the final design since opportunities for teamwork cannot always be assured in the rural setting. It was also important to choose attributes and levels that participants were likely to trade off. In other words, we sought to create a situation where participants would be prepared to accept a higher level of one attribute over another (i.e. very flexible work hours traded off against a lower level of remuneration). Figure 1 illustrates the themes that emerged and the attributes derived from the themes.

### Step 2—Setting levels for each factor (attribute)

Following the identification of the six attributes and the most salient factor for each, there was a need to determine appropriate attribute “levels” or categories within each factor. The prevailing conditions for AHPs working in the disability sector were chosen as base levels for each attribute. Additional levels were then determined and were intended to represent a reasonable improvement from the base level. As noted by Al-Janabi et al. [31], when setting levels, it is important to come to a balance between conciseness and sensitivity. It is important that the levels of the attributes represent realistic choices. There is also a need to consider the burden for respondents [19]. Usually two to three levels per attribute provides an appropriate number of choices for the final questionnaire.

After the attributes and levels were identified, the study reference group was asked to comment on the wording and applicability of the levels. The reference group’s feedback, further team discussion, and reference to current policy in the sector were incorporated into the wording of the DCE attributes and levels.

In this study, three levels were selected for each attribute (see Table 2). It was particularly difficult to define qualitative levels for attributes in this study (i.e. minimal access to professional development, adequate access to professional development). Non-prescriptive examples of each level of these attributes were therefore developed, which respondents could refer to while completing the survey.

**Table 2:** **Final attributes and levels**

**Attribute**	**Definition**	**Levels**	**Examples**
Travel arrangements	Travel that requires overnight stays away from home	One or less nights away per month	
		Two or three nights away per month	
		Four or more nights away per month	
Flexibility	Ability to negotiate your hours of work	Little or no flexibility in work hours	• Management requires fixed start and finish times
		Some flexibility in work hours	• Management is open to negotiation on occasional variation to hours of work
		Very flexible work hours	• Management is open to regular variation to hours of work
Professional support	Profession-specific advice and support	Rarely	• Hardly ever available even when needed
		Sometimes	• Available but not always as needed
		Readily	• Available immediately as needed
Professional development (PD)	Opportunity to undertake *formal* professional development activities	Minimal	• Occasional access to formal PD
		Adequate	• Access to formal PD to maintain skills in core area of practice
		Ideal	• Access to formal PD to maintain and develop skills in a range of relevant areas
Remuneration	Rural salary loading above current salary	5% above your current salary	
		10% above your current salary	
		15% above your current salary	
Autonomy of practice	Freedom to use professional judgement	Limited capacity for independent professional decision making	• Freedom to make independent professional decisions the minority of the time
		Some level of independent professional decision making	• Freedom to make independent professional decisions around half the time
		High level of independent professional decision making	• Freedom to make independent professional decisions the majority of the time

## Discussion

### Summary of findings

From an initial group of eight themes, an inductive data analysis process involving consultation with stakeholders (i.e. via the project management and reference groups) and policy analysis helped to derive the final set of six attributes and their levels. These attributes were 1) travel arrangements, 2) flexibility in work hours, 3) professional support, 4) professional development, 5) autonomy of practice, and 6) remuneration.

### Design of DCE questionnaire

The selection of attributes and levels are important elements of the design of DCEs. However, as described by some authors, DCE studies provide limited information on how the final set of attributes was derived [31,32]. The results from this study allowed us to empirically derive a set of attributes and levels of workforce retention for AHPs working with people with disability in rural and remote areas. These attributes and levels will be used to generate hypothetical job scenarios and then used in a DCE that will explore AHPs’ preferences as part of a broader survey on retention issues. In the design of DCEs, the selection of attributes and the levels of each attribute are important elements. As Ryan noted, “*attributes should be important to service users* [in this case AHPs who are employed under the policies] *and policy makers with levels being “plausible” and capable of being traded*” [33]. In this study, information from the focus groups and interviews, the policy and literature review, and stakeholders’ input (via the project management and reference groups) were integral to defining the attributes and levels.

### Comparison with the literature on factors

Even though this is the first attempt at developing a DCE to explore preferences of AHPs working with people with disability in rural and remote areas, retention factors have been explored via surveys and qualitative studies. Not surprisingly, some of the themes that emerged from this qualitative study are similar to those described by studies of AHPs working in rural areas [4,34,35]. Authors of a survey of rural allied health workforce in NSW concluded that important retention factors included the following: the availability of flexible employment opportunities, improved opportunities for acquiring new knowledge and skills, and career advancement. They also highlighted the importance of autonomy in rural practice [36].

It is important to note that the above-mentioned survey included 21 different allied health occupations and did not focus on disability [36]. Professional (remuneration, lack of professional development, little professional support or recognition) and personal (family-related) factors have also been described in the literature [36-38]. Participants in the current study described the importance of access to professional networks and improved supervision, especially for new graduates. Denham and Shaddock identified opportunity for professional development and professional support or recognition as important professional factors in retaining AHPs who work in rural areas with people with developmental disability [39].

In our study, participants found overnight stays problematic. This is a dimension of travel that to date has not been explored. The literature in relation to the amount of travel done by AHPs working in rural areas is limited. Wielandt and Taylor, in Canada, reported that travel was described by some rural occupational therapists as rewarding. However, the majority identified it as a challenge (mainly due to the hazardous driving conditions in winter) [40]. Bent, in Australia, found that despite travelling vast distances, rural AHPs working in health in central Australia thought travelling was a good opportunity to see the outback scenery [41].

Gillham and Ristevski [42] described financial incentives as an important recruitment, not retention, factor. Remuneration, however, as depicted by the participants in our study is an important factor when deciding to continue to work with people with disability in rural areas. Differences across sectors (government/non-government, disability/health) were described and appeared important. For example, Mangham and Hanson [18] explored the employment preferences of public sector nurses in Malawi. In this study, salary enhancement improved motivation and retention of nurses and could potentially reduce the high rates of attrition in the public sector [18].

Even though research to date has identified a range of factors associated with retention of AHPs, it provides only weak evidence on the relative importance of these factors. To date, there are no published studies on employment preferences of AHPs working with people with disability in rural areas.

### Comparison with other qualitative DCE development studies

In an effort to improve the face validity of DCE attributes and levels, qualitative interviews are increasingly being used in their creation [31,32,43-46]. The qualitative methods used in this study are similar to those used in other DCE development qualitative studies. This study is distinctive in that it complemented the information from the interviews and focus groups [10] with a policy document review [25,30] and support from the project management and reference groups that involved different stakeholders. The iterative process allowed refinement of the language to make it meaningful to potential survey participants. Feedback from stakeholders and the results from the policy review attempted to make the attributes “manipulable” by policy [32].

### Strengths and limitations

Recognition of the value of qualitative research methods to develop DCE attributes has been growing [32]. However, as noted by Coast and Harrocks [47], there is an inherent tension between conducting qualitative research to gain a deep understanding of a particular issue and reducing data to describe a small number of attributes for the purpose of further investigation through a DCE format. As “brief descriptions of attributes and their levels could never do justice to the complexity of individuals’ preferences” (p29). In this study, we benefitted from the richness of a full qualitative data set as reported in other publications [10,25] by embedding the DCE development in a larger qualitative study that explored not only workforce issues for AHPs working in disability and living in a rural area, but also policy issues and service delivery pathways [26].

## Conclusion

The use of different qualitative research methods allowed the development of a policy-relevant DCE questionnaire that was grounded in the experience of the target population (AHPs).
